# Tonsil mesenchymal stem cells-derived extracellular vesicles prevent submandibular gland dysfunction in ovariectomized rats

**DOI:** 10.18632/aging.203947

**Published:** 2022-03-13

**Authors:** Ji Min Kim, Jeong Hun Kim, Keunyoung Kim, Sung-Chan Shin, Yong-Il Cheon, Hyung Sik Kim, Jin-Choon Lee, Eui-Suk Sung, Minhyung Lee, Gi-Cheol Park, Byung-Joo Lee

**Affiliations:** 1 Pusan National University Medical Research Institute, Pusan National University School of Medicine, Pusan National University, Busan, Republic of Korea; 2 Biomedical Research Institute, Pusan National University Hospital, Busan, Republic of Korea; 3 Department of Nuclear Medicine and Biomedical Research Institute, Pusan National University Hospital, Busan, Republic of Korea; 4 Department of Otorhinolaryngology-Head and Neck Surgery, Pusan National University School of Medicine, Pusan National University, Busan, Republic of Korea; 5 Department of Life Science in Dentistry, School of Dentistry, Pusan National University, Yangsan, Republic of Korea; 6 Institute for Translational Dental Science, Pusan National University, Yangsan, Republic of Korea; 7 Department of Otorhinolaryngology-Head and Neck Surgery, Pusan National University School of Medicine and Biomedical Research Institute, Pusan National University Yangsan Hospital, Yangsan, Gyeongnam, Republic of Korea; 8 Department of Otolaryngology-Head and Neck Surgery, Samsung Changwon Hospital, Sungkyunkwan University School of Medicine, Changwon, Republic of Korea

**Keywords:** tonsil mesenchymal stem cell, extracellular vesicles, ovariectomy, xerostomia, menopause, salivary gland

## Abstract

Dry mouth that occurs after menopause significantly reduces the quality of life of the elderly. The extracellular vesicles derived from mesenchymal stem cells are being studied for application in various pathological conditions in the field of tissue regenerative medicine. This study is to investigate the therapeutic effect on salivary gland dysfunction occurring after ovariectomy using tonsil mesenchymal stem cells (T-MSCs)-derived extracellular vesicles. The rats were divided into the following groups: sham-operated rats (SHAM), rats that underwent ovariectomy (OVX), and rats that underwent OVX surgery and were simultaneously injected with T-MSC-derived extracellular vesicles (OVX+EV). The rats were sacrificed 6 weeks after ovariectomy. Estradiol levels decreased in the OVX group compared with those in the SHAM group. Extracellular vesicles had no effect on estradiol levels or estrogen receptor β expression. The evaluation of pro-inflammatory cytokines, TNF-α and IL-6, increased in the OVX group and decreased in the OVX+EV group. The expressions of collagen I and TGFβI increased in the OVX group but decreased in the OVX+EV group. Moreover, to examine submandibular gland function, AQP5 and α-amylase expressions were downregulated in the OVX group, but improved upon exosome injection. In conclusion, T-MSC-derived extracellular vesicles are useful for the prevented submandibular gland dysfunction that occurs after menopause.

## INTRODUCTION

Menopause is a physiological process characterized by the cessation of menstruation, resulting from a loss of ovarian function. Menopausal changes can induce vasomotor symptoms, such as hot flushes, night sweats, and dyspareunia, as well as dry eyes and dry mouth [1]. Menopause is accompanied by physical and functional changes in the oral cavity. Previous studies have revealed that these changes are caused by postmenopausal hormone deficiency, which could induce a reduction in salivary gland secretions [2]. Menopause-induced xerostomia induces dry mouth, which is associated with oral pain and difficulties in speech. [3, 4]. Therefore, xerostomia reduces the quality of life of women after menopause. Studies on the mechanism of reduction of salivation after menopause are important for the prevention of oral diseases and salivary gland dysfunction. However, the mechanism associated with the reduction of salivary gland function during menopause has not been well studied.

Mesenchymal stem cells (MSCs) have great therapeutic potential because of their ability to self-renew and differentiate into multiple tissues. MSCs are multipotent cells that can self-renew and differentiate into several cell types of the same mesenchymal lineage, including osteocytes, adipocytes, and chondrocytes [5, 6]. The proliferation and differentiation potential of MSCs are used in several tissue engineering and regenerative medicine fields, including in studies on radiation-induced salivary gland dysfunction [7]. Recently, human palatine tonsils have emerged as a new source of mesenchymal stem cells (T-MSCs) [8, 9]. T-MSCs contributed to the attenuation of excessive inflammation in surgical lesions, as well as to the augmentation of epidermal and dermal regeneration [10]. Compared with other MSCs, T-MSCs can be more easily obtained from the palatine tonsil tissue through tonsillectomy, the most commonly performed surgery in otolaryngology. In our previous study, we found that the proliferation potential of T-MSCs, which is the most important functional factor of stem cells, was significantly higher than that of bone marrow-derived MSCs or adipose tissue-derived MSCs [11]. Therefore, we assessed the therapeutic effects of T-MSCs on menopause-induced xerostomia.

MSCs are a good candidate for cell therapy in tissue engineering and regenerative medicine, and the therapeutic function of MSCs can be attributed mainly to their paracrine effect [12]. Extracellular vesicles containing exosomes derived from MSCs have paracrine effects [13]. Differentiation of engrafted MSCs into unwanted cells, such as osteocytes and chondrocytes, and the induction of immune responses by allogeneic MSCs transplantation have been reported [14–16]. Therefore, the safety of allogeneic MSCs transplantation is still controversial [17].

Exosomes are included in extracellular vesicles that are generated inside the cell and released to the outside that are small membrane particles (40–150 nm in size) of endosomal origin. They play crucial roles in cell-cell communication and mediate gene expression by delivering miRNAs, mRNAs, and proteins to recipient cells [18, 19]. Recently, exosomes have been recognized as paracrine factors that are released by almost all cell types, and there are studies on their application for the treatment of various diseases in several organs [20–23]. T-MSCs has high proliferation ability compared to other MSCs [9]. It is expected that the constituents of the isolated extracellular vesicles will differ depending on the type of MSCs, but there are still no studies on the differences between T-MSCs and extracellular vesicles derived from other MSCs. Further, we plan to study the differences in exosomes isolated from tonsil MSC cells or other cells. Moreover, there are no studies on the therapeutic or preventive effects of extracellular vesicles on various symptoms caused by menopause, especially xerostomia. Therefore, in order to investigate the therapeutic effect of T-MSCs-derived extracellular vesicles on menopause-induced xerostomia, we evaluated the morphological and functional changes in the submandibular gland by injecting T-MSCs-derived extracellular vesicles into ovariectomized rats.

## RESULTS

### Characteristics and differentiation of T-MSCs

**Figure 1:**
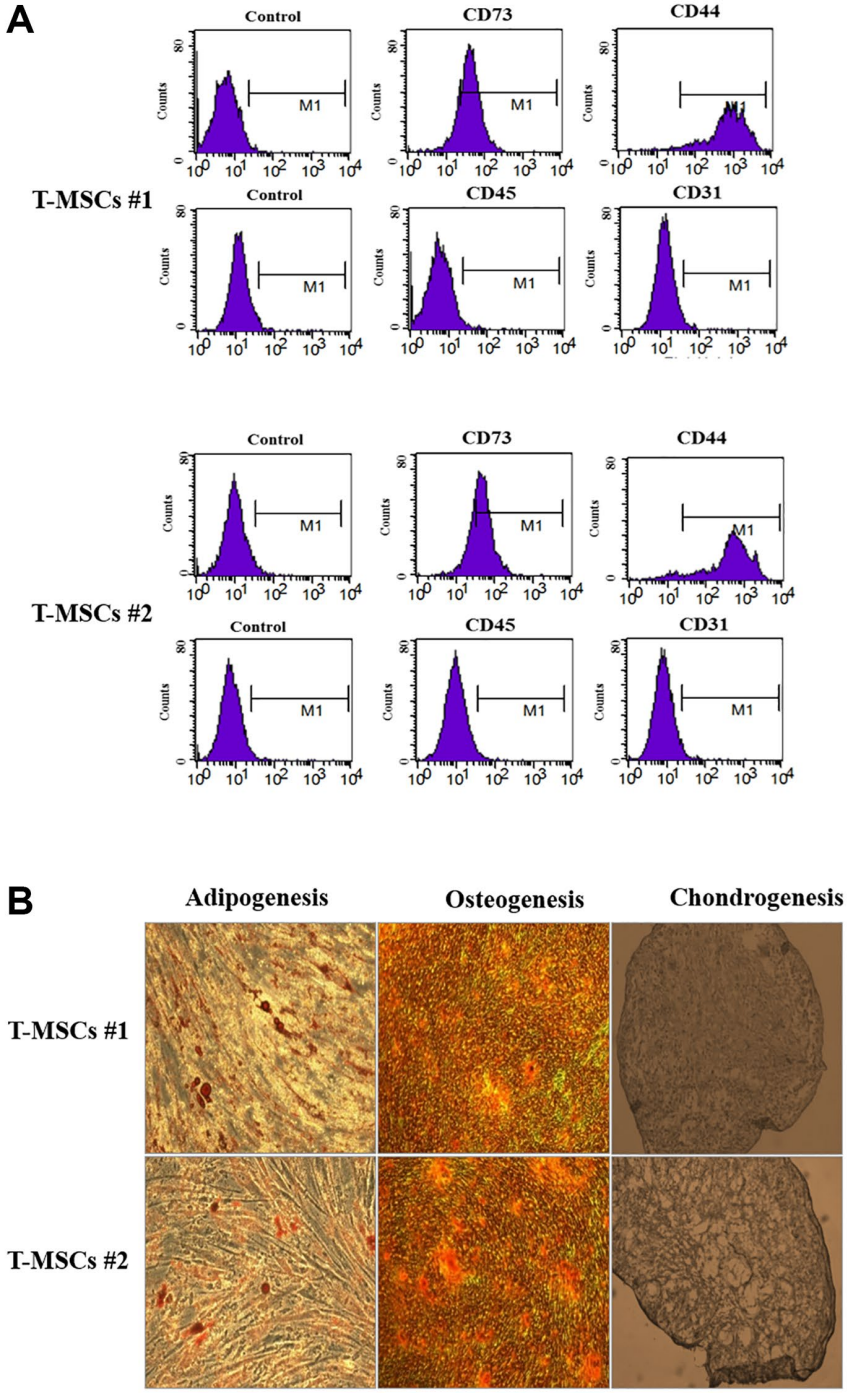
**Characterization of human tonsil mesenchymal stem cells (T-MSCs).** Surface marker profiling determined by fluorescence-activated cell sorting in T-MSCs. The cells were positive for CD73 and CD44 and negative for CD45 and CD31; the upper panel is T-MSCs #1 and the lower panel is T-MSCs #2 (**A**). The adipogenesis, chondrogenesis, and osteogenesis of T-MSCs were examined by oil red staining, alizarin red staining, and Alcian blue staining, respectively; the upper panel is T-MSCs #1 and the lower panel is T-MSCs #2 (**B**).

The characteristics of human T-MSCs are shown in Figure 1. The results of flow cytometry showed that both T-MSC#1 (Figure 1A, upper panel) and T-MSC#2 (Figure 1A, lower panel) were positive for CD73 and CD44, but negative for CD34 and CD45. In addition, we verified the characteristics of MSCs by differentiating the cells, and we determined adipogenesis, osteogenesis, and chondrogenesis in the differentiated T-MSCs (Figure 1B).

### Identification of T-MSCs-derived extracellular vesicles

**Figure 2:**
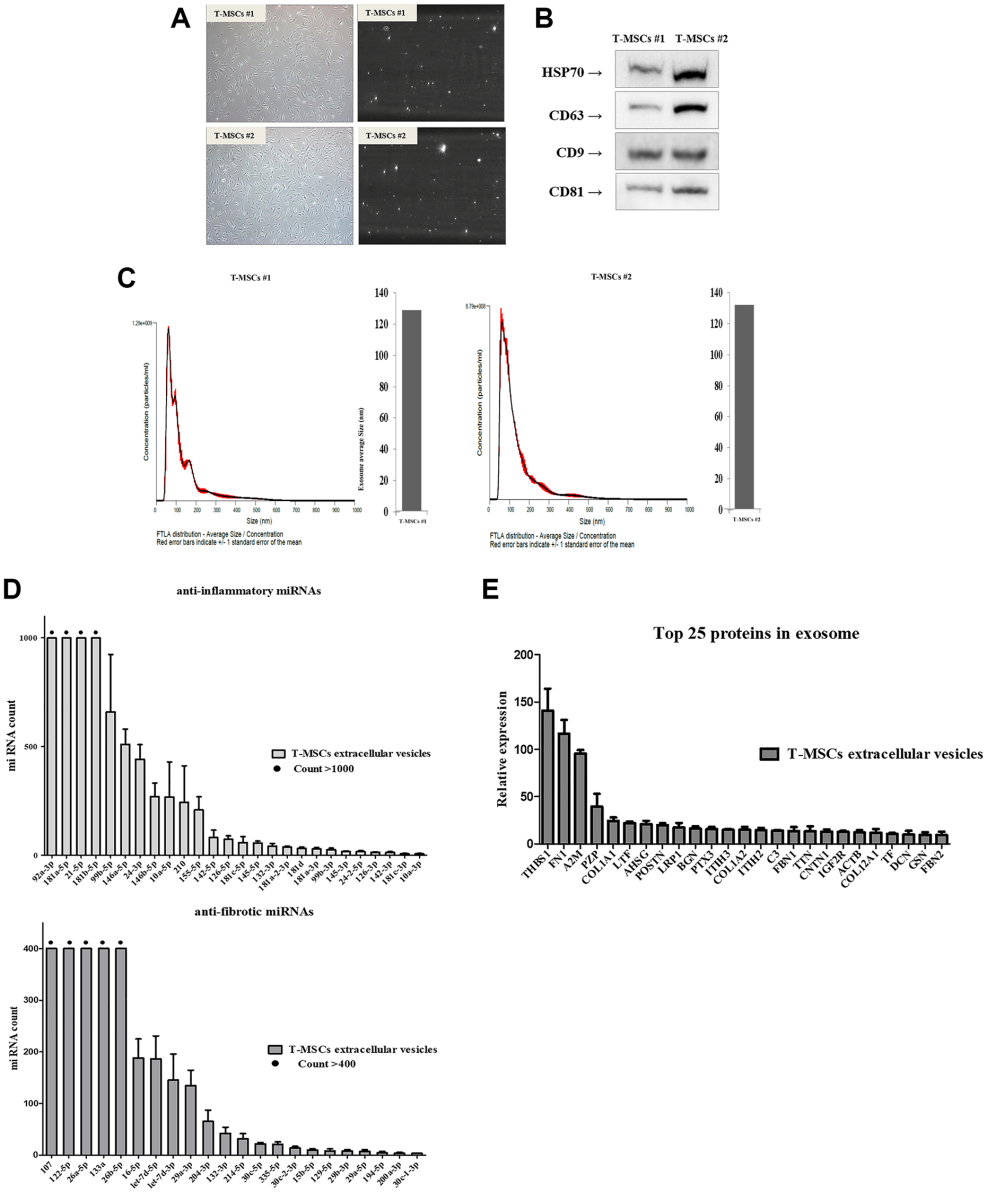
**Identification of T-MSCs derived-extracellular vesicles.** Nanoparticle tracking analysis of T-MSCs derived-extracellular vesicles. Normal cell (left) and screen shot from video of light scatter of placental vesicles overlaid with a graph of vesicle size and concentration, as determined by nanoparticle tracking analysis (right) (**A**). Western blot illustrating the characteristic surface markers of extracellular vesicles, HSP70, CD63, CD9, and CD81, present in the extracellular vesicles (**B**). Histogram of the nanoparticle tracking analysis demonstrating the size distribution of T-MSCs-derived extracellular vesicles after isolation through centrifugation (**C**). The presence of microRNA (miRNAs) in T-MSC-derived extracellular vesicles. T-MSCs-derived exosomes contain approximately 26 anti-inflammatory miRNAs and approximately 22 anti-fibrotic miRNAs (**D**). T-MSCs-derived extracellular vesicles contain 25 proteins (**E**).

The ultrastructure and size distribution of extracellular vesicles derived from T-MSCs #1 (Figure 2A, upper panel) and T-MSCs #2 (Figure 1A, lower panel) were determined by conducting a nanoparticle tracking analysis. First, 10 nm gold particles were immersed in water and visualized with a 532 nm (green) laser of a NanoSight instrument. The video shows the light scattered from the particles as they move under Brownian motion. The nanoparticles were small, and hence, the particles were quite dim and moved very quickly (Figure 2A, Supplementary Videos 1 and 2). Next, we identified purified T-MSCs-derived extracellular vesicles through western blotting. The surface markers, HSP70, CD63, CD9 and CD81, were detected in both T-MSCs #1 and T-MSCs #2-derived extracellular vesicles (Figure 2B). Analysis of the size and concentration of isolated T-MSC-derived extracellular vesicles using NTA demonstrated a bell-shaped curve, with the majority of the area under the curve falling within the characteristic extracellular vesicles size range of 50~150 nm. The FTLA distribution values (average size/concentration) for T- MSCs #1-derived extracellular vesicles were 130.4 ± 2.1 nm and T-MSCs #2-derived extracellular vesicles were 132.3 ± 2.2 nm, respectively (Figure 2C). Generally, the size range of extracellular vesicles is 50~150 nm. The T-MSCs-derived extracellular vesicles were extracellular vesicles in size and properties. The total extracellular vesicles concentrations for T-MSCs #1-derived extracellular vesicles were 9.22e + 010 ± 7.64e + 008 particles/ml and T-MSCs #2-derived extracellular vesicles were 7.40e + 010 ± 1.60e + 009 particles/ml. We also investigated microRNA and protein analysis of T-MSC-derived extracellular vesicles. The presence of large amounts of small RNAs in T-MSCs-derived extracellular vesicles suggests that extracellular vesicles may contain microRNAs (miRNAs). Therefore, NGS analysis was used to identify the miRNAs in the T-MSC-derived extracellular vesicles. The results showed that the T-MSC-derived extracellular vesicles carried approximately 26 anti-inflammatory miRNAs, such as 92a-3p, 181a-5p, 21-5p, and 181b-5p, and approximately 22 anti-fibrotic miRNAs, including 107, 122-5p, 26a-5p, 133a, and 26b-5p (Figure 2D). Moreover, proteomics analysis was used to identify the proteins in the T-MSCs-derived extracellular vesicles. Results indicated that T-MSCs-derived extracellular vesicles carried 25 proteins, and the most abundant protein was THBSI (Figure 2E).

### Serum levels of estradiol and the expression of estrogen receptor β

**Figure 3:**
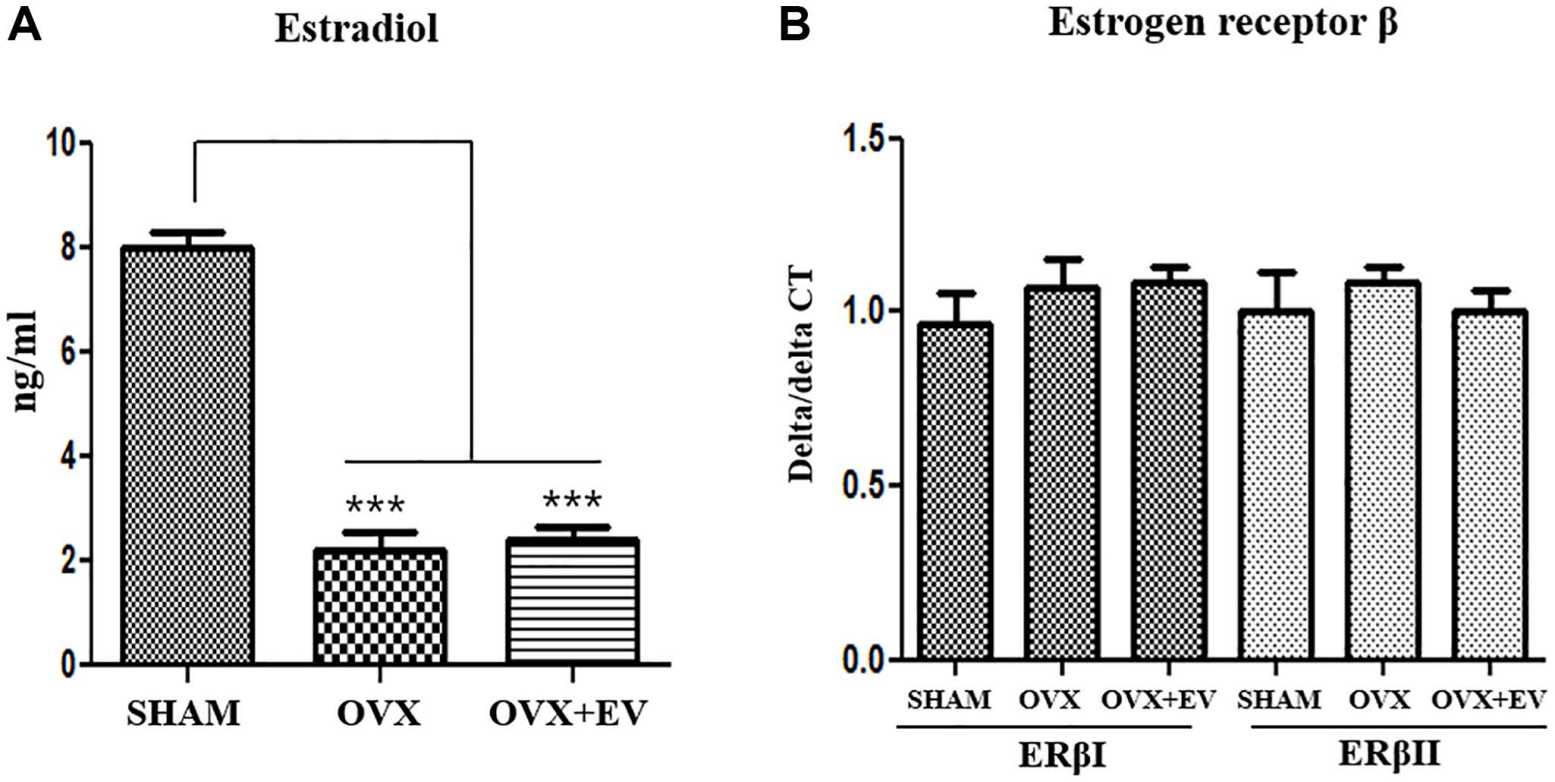
**Serum estradiol levels and expression of estrogen receptor β.** Serum estradiol levels were lower in the OVX group than in the SHAM group. Exosome treatment did not affect the serum estradiol levels (**A**). Quantitative polymerase chain reaction analysis of genes encoding representative estrogen receptor β in the submandibular gland. The expression of ERβI and ERβII did not change between the groups (**B**). One-way ANOVA test; ^***^p < 0.001 vs. SHAM.

We determined the levels of estradiol and the expression of estrogen receptors in ovariectomized rats. The result shows the changes in the serum levels of estradiol (Figure 3A). The level of serum estradiol in the OVX group was lower than that in the SHAM group. However, the estradiol concentration in the OVX group was similar to that observed in the OVX+EV group. To evaluate the effect of sex hormones on sex hormone receptors, we performed qPCR analysis to determine the expression levels of two isoforms of estrogen receptor, ERα and ERβ, in the submandibular gland. In our previous study, we could not detect the expression of ERα in the submandibular gland of female rats with qPCR [24]. Hence, in this study, qPCR was conducted to evaluate the expression levels of ERβI and ERβ. The expression levels of ERβI and ERβII were not significantly different between the groups (Figure 3B). Ovariectomy led to a significant reduction in the amount of estradiol, and EXO had no effect on both serum estradiol levels and ERβ expression.

### Secretion of pro-inflammatory cytokines

**Figure 4:**
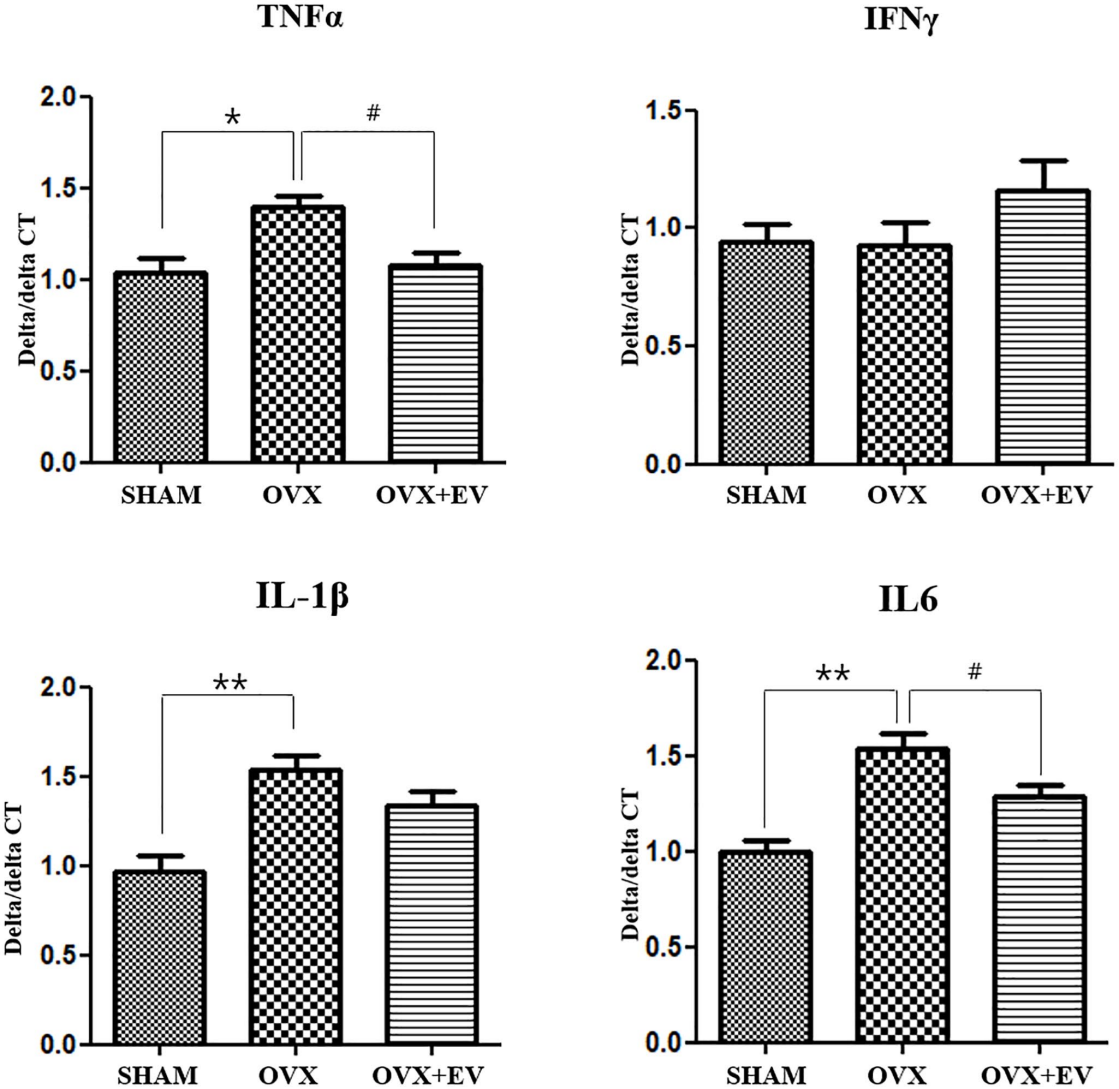
**Anti-inflammatory effect of T-MSCs-derived extracellular vesicles.** Quantitative polymerase chain reaction analysis of tumor necrosis factor alpha (TNF-α), interferon γ, interleukin 1β (IL-1 β), and interleukin 6 (IL-6) in the submandibular gland. The mRNA expression of TNF-α, IL-1, and IL-6 was higher in the OVX group than in the SHAM group. Extracellular vesicles treatment decreased the TNF-α and IL-6 levels. One-way ANOVA test; ^*^p < 0.05 and ^**^p < 0.01 vs. SHAM, ^#^*p* < 0.05 vs. OVX.

The levels of tumor necrosis factor alpha (TNF-α, *p* < 0.05), interleukin 1β (IL-1 β, *p* < 0.01), and interleukin 6 (IL-6, *p* < 0.01) significantly increased in the OVX group, compared with the SHAM group. However, the OVX+EV group showed the decreased levels of pro-inflammatory cytokines (TNF-α, *p* < 0.05; IL-6, *p* < 0.05). In addition, there were no significant differences in the levels of interferon γ (IFN-γ) between the groups. The results also revealed that the secretion of pro-inflammatory cytokines was significantly increased in the OVX groups, but upon the injection of extracellular vesicles, the levels of cytokines and inflammatory response decreased in the submandibular gland of ovariectomized rats (Figure 4).

### Improvement of fibrosis

**Figure 5:**
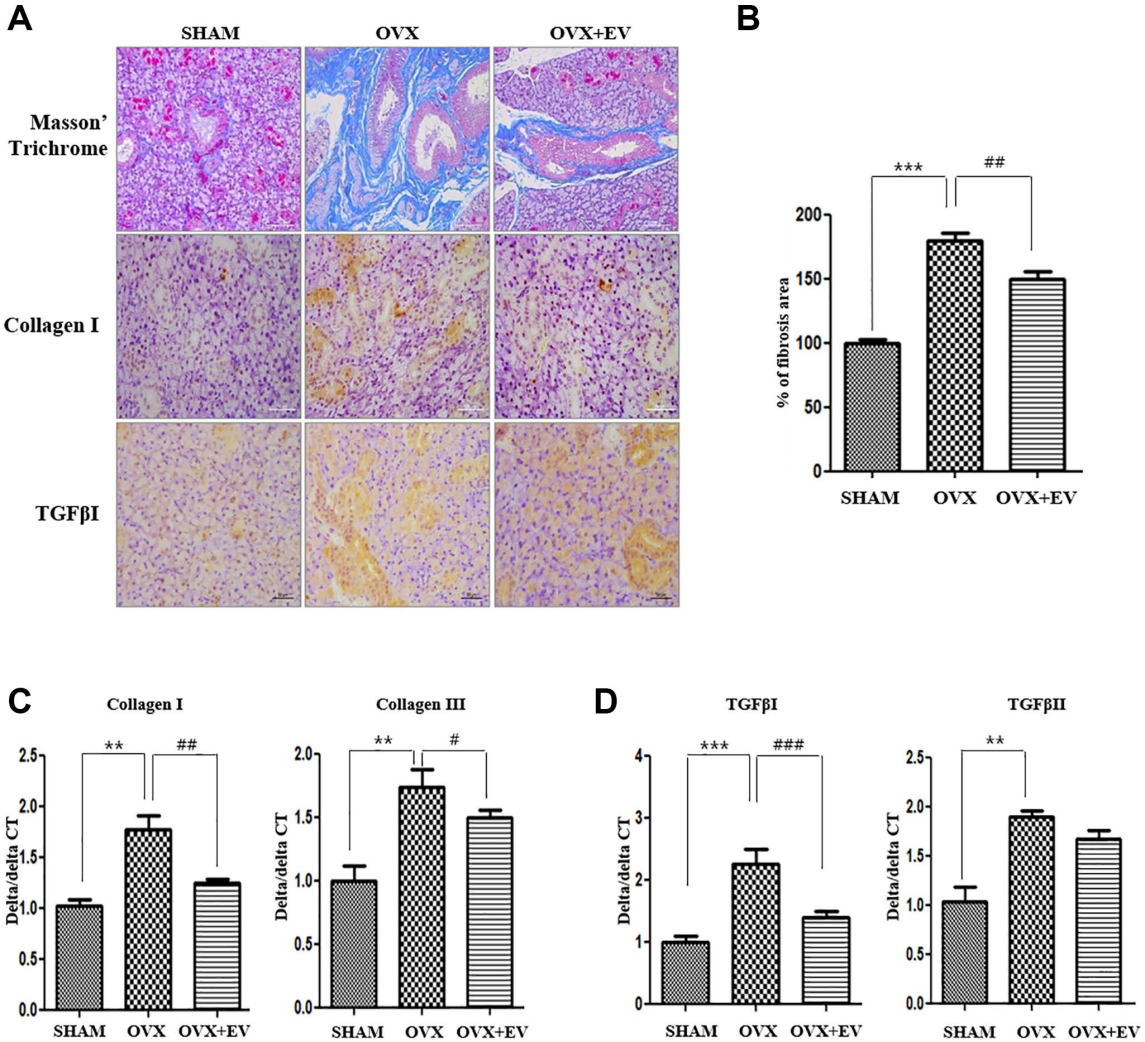
**Anti-fibrotic effect of T-MSCs-derived extracellular vesicles.** In the results of morphometric analysis, the fibrotic area was significantly higher in the OVX groups than in the SHAM group. The OVX+EV group showed decreased collagen type I and TGF-βI expression. Quantitative polymerase chain reaction (qPCR) analysis of collagen I and III in the submandibular gland (**A** and **B**). The mRNA expression of collagen I and III increased in the OVX group and decreased mRNA expression of collagen I and III in the OVX+EV group, compared with that in the SHAM group (**C**). qPCR analysis of TGF-β1 and TGF-βII in the submandibular gland. The mRNA expression of TGF-β1 and TGF-βII increased in the OVX group and decreased mRNA expression of TGF-β1 in the OVX+EV group, compared with that in the SHAM group (**D**). One-way ANOVA test; ^**^p < 0.01 and ^***^p < 0.001 vs. SHAM. ^##^*p* < 0.01 and ^###^*p* < 0.001 vs. OVX.

The results of MT staining showed that the degree of fibrosis in the submandibular gland tissue was significantly higher in the OVX group (*p* < 0.001) than that in the SHAM group. However, the OVX+EV group showed significantly decreased fibrosis (*p* < 0.01) (Figure 5A upper panel and Figure 5B). The collagen I deposition increased in the OVX group, and decreased by extracellular vesicles injection. We also confirmed that the mRNA expression of collagen I and collagen III in the OVX group was significantly higher (*p* < 0.01) than that in the SHAM group. However, the OVX+EV group showed significantly decreased mRNA expression of collagen I (*p* < 0.01) and III (*p* < 0.05) (Figure 5C). After extracellular vesicles treatment, TGF-β1 returned to levels comparable to those in the SHAM group (Figure 5A, bottom panel). In addition, the mRNA expression of TGF-β1 (*p* < 0.001) and TGF-βII (*p* < 0.01) was significantly upregulated in the OVX group, and the OVX+EV group showed significantly decreased expression of TGF-β1 (*p* < 0.001) (Figure 5D).

### Recovery of salivary gland dysfunction

**Figure 6:**
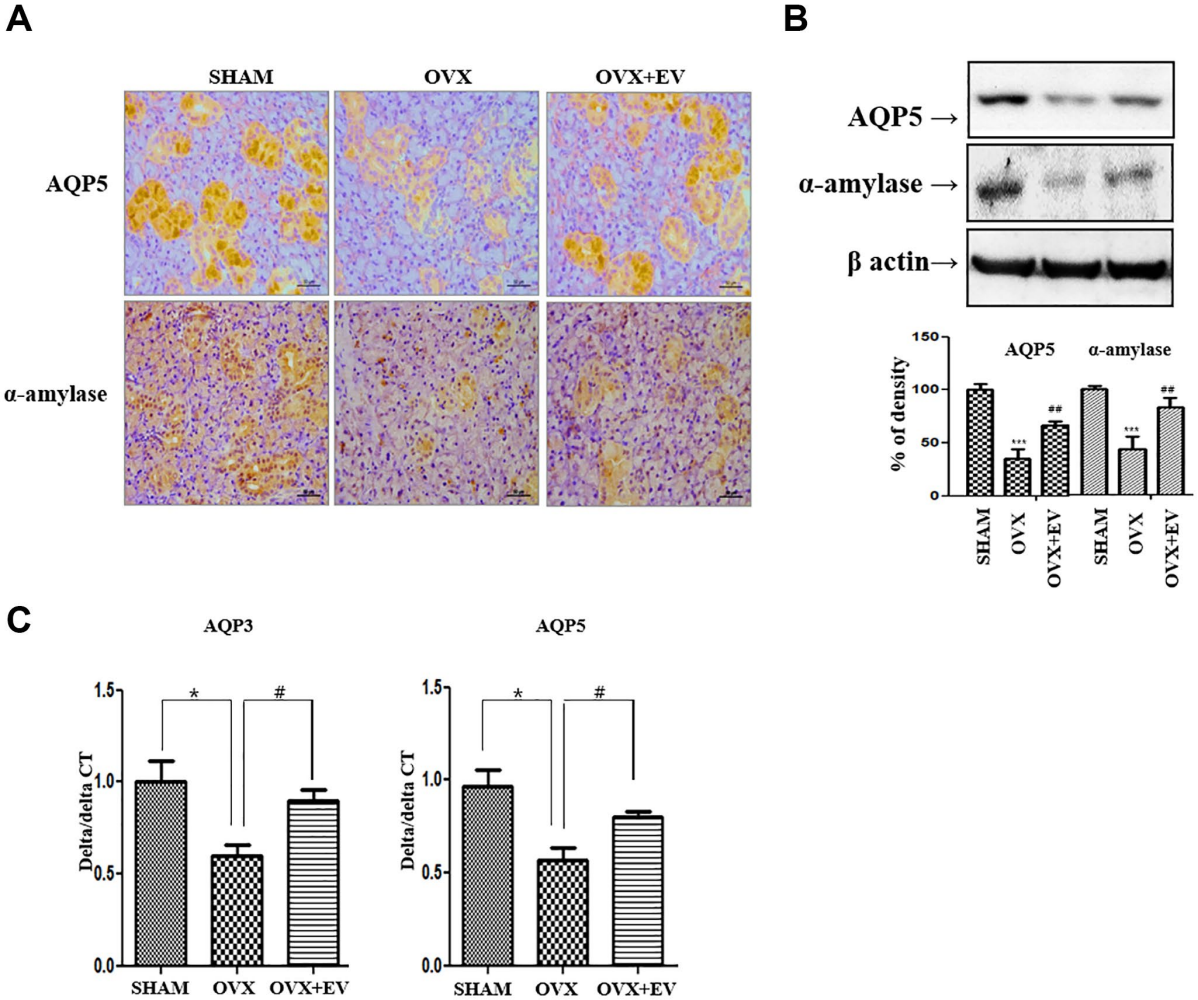
**Increased salivary gland function by injected T-MSCs-derived extracellular vesicles.** The AQP5- and α-amylase-positive areas significantly decreased in the OVX groups compared with those in the SHAM group. However, AQP5 and α-amylase expression increased in the OVX+EV treatment group (**A** and **B**). The mRNA expression of AQP3 and AQP5 decreased in the OVX group and increased mRNA expression of AQP3 and AQP5 in the OVX+EV treatment group, compared with that in the SHAM group (**C**). One-way ANOVA test; and ^*^p < 0.05 and ^***^p < 0.001 vs. SHAM, ^#^*p* < 0.05 and ^##^*p* < 0.01 vs. OVX.

To determine if T-MSCs-derived extracellular vesicles improved the function of the submandibular gland, we investigated the expression of AQP5 and α-amylase. The immunostaining and protein expression revealed that AQP5 (middle panel) and α-amylase (down panel) were significantly lower in the OVX group than in the SHAM group (Figure 6A). However, the injection of T-MSCs-derived extracellular vesicles increased the staining densities of AQP5 and α-amylase, relative to those observed in the OVX group. According to the results of western blot analysis, the expression of AQP5 and α-amylase was significantly lower in the OVX group than in the SHAM group (*p* < 0.001) (Figure 6B). The expression of AQP5 and α-amylase increased in the OVX+EV group (*p* < 0.01). We also confirmed that the mRNA expression of the representative aquaporin subtypes involved in saliva secretion, AQP3 and AQP5, was significantly lower in the OVX group (*p* < 0.05) than in the SHAM group. However, the OVX+EV group showed significantly increased mRNA expression of AQP3 and AQP5 (*p* < 0.05) (Figure 6C). These results indicate that T-MSCs-derived extracellular vesicles can reverse the effect of ovariectomy and improve the functions of the submandibular gland.

## DISCUSSION

Menopause-induced xerostomia greatly affects the quality of life of postmenopausal women. There is no standard effective treatment for salivary gland dysfunction that occurs after menopause. There has been an increase in elderly population, and hence, the quality of life of the elderly has become important. Studies on xerostomia using MSCs derived from bone marrow or umbilical cord have been reported, but there are no reports of the use of MSCs-derived exosomes [25, 26]. The safety of allogeneic MSCs is still controversial. Several studies have used exosomes instead of MSCs to study multiple tissue regeneration [27, 28], but there are no studies on their use for menopause-induced xerostomia.

This is the first study to investigate the usefulness of MSCs-derived exosomes for the improvement of salivary gland dysfunction that occurs after ovariectomy. Various MSCs have been clinically studied in tissue engineering or regenerative medicine, but there is still controversy over the immunological safety of allogenic MSCs transplantation [17, 29]. Therefore, many recent studies have involved the use of exosomes, which, unlike MSCs, cannot cause pulmonary embolization and cannot cross the blood brain barrier due to their size (40–150 nm) [30]. Exosomes are enriched in many bioactive molecules, such as lipids, proteins, mRNA, transfer RNA, long noncoding RNA, miRNA, and mitochondrial DNA [31]. Extracellular vesicles containing exosomes are crucial messengers that are present in biological fluids and are involved in multiple physiological and pathological processes [32]. MSCs-derived exosomes have shown promising therapeutic effects in several tissues or organs through the regulation of angiogenesis, anti-inflammation, anti-fibrosis, anti-apoptosis, and immunomodulation [33–36]. The NGS results of the extracellular vesicles of T-MSCs showed the presence of anti-inflammatory miRNAs, such as 92a-3p, 181a-5p, 21-5p, and 181b-5p. In addition, miRNAs involved in anti-fibrosis, such as 107, 122-5p, 26a-5p, 133a, and 26b-5p, were also observed. In addition, THBSI, which has an anti-inflammatory effect, was the most abundant protein in extracellular vesicles of T-MSCs.

The exact mechanism of menopause-induced xerostomia has remained unclear. The induction of ferroptosis in the submandibular gland of ovariectomized rats, which increases inflammatory cytokine secretion and fibrosis in the submandibular gland, has been reported as an important mechanism associated with salivary dysfunction [37]. In this study, the secretion of inflammatory cytokines and fibrosis increased in the submandibular gland after ovariectomy. In addition, our results showed that salivary gland dysfunction occurred through decreased expression of AQP and amylase. Upon the injection of T-MSCs-derived extracellular vesicles, the secretion of inflammatory cytokines and fibrosis in the submandibular gland decreased and the expression of aquaporin and amylase increased. These findings indicate that T-MSCs-derived extracellular vesicles can prevent postmenopausal salivary gland dysfunction by anti-inflammation and anti-fibrosis mechanisms. In a recently published chronic liver disease model study, it was reported that T-MSCs-derived small extracellular vehicles had an anti-fibrotic effect, which was similar to our study [38].

In this study, TNFα, IL-1β, and IL-6 levels increased significantly in the submandibular gland tissue after ovariectomy. However, when T-MSCs-derived extracellular vesicles were injected, TNFα and IL-6 levels significantly decreased in the salivary gland. In a previous study, adipose tissue-derived autologous MSCs-derived exosomes attenuated renal inflammation, decreased TNF-α, IL-6, and IL-1-β in the renal vein [22], and reduced the serum levels of TNF-α, IFN-γ, IL-6, IL-18, and IL-1β in a hepatitis model [39, 40]. These findings are consistent with the result of this study, and they indicate that MSCs-derived exosomes reduce inflammation in several disease models.

The expression of fibrosis-associated collagen I, TGF-βI and TGF-βII increased in the submandibular gland after ovariectomy. However, when T-MSCs-derived extracellular vesicles were injected, the expression of collagen I and TGF-β I decreased significantly and fibrosis decreased generally. Human umbilical cord MSCs ameliorated liver fibrosis by inhibiting both the epithelial-mesenchymal transition of hepatocytes and collagen production. Similarly, exosomes derived from human umbilical cord MSCs increased renal capillary density and reduced fibrosis in rats with renal ischemia-reperfusion injury [41]. Moreover, human umbilical cord MSCs-derived exosomes reduced TGF βI in an animal model of liver injury [42]. These findings are similar to the results of this study, and they indicate that MSC-derived exosomes can reduce fibrosis in various disease models.

Inflammation and fibrosis can impair the functions of the salivary gland. The salivary gland hypofunction that occurs with Sjögren’s syndrome is associated with an increase in TNF-α. An increased level of TNF-α cause a reduction in the expression of AQP 5, resulting in salivary gland hypofunction [43, 44]. However, treatment with TNF-α is not effective for salivary gland hypofunction. Some conditions, such as menopause and Sjögren’s syndrome, are associated with various cytokines. Therefore, the use of extracellular vesicles with various anti-inflammatory and anti-fibrotic functions can exert enhanced therapeutic effect in diseases involving various cytokines.

In the current study, T-MSCs-derived extracellular vesicles alleviated salivary gland dysfunction caused by menopause through anti-inflammatory and anti-fibrosis mechanisms. T-MSCs-derived extracellular vesicles contain many miRNAs and proteins involved in the mechanism of inflammation and fibrosis. Through the interaction of these molecules, T-MSCs-derived extracellular vesicles can exert preventive and therapeutic effects on salivary gland dysfunction that occurs after ovariectomy. In the absence of an effective standard treatment for menopause-induced xerostomia, T-MSCs-derived extracellular vesicles may be considered as an alternative method. Further studies involving extracellular vesicles are necessary to identify miRNAs and proteins that play important roles in the impairment of salivary gland function after menopause.

## METHODS

### Isolation and characterization of T-MSCs

T-MSCs were isolated from human tonsil tissue, as described in our previous studies [19]. For this study, palatine tonsils were obtained from two different patients diagnosed with chronic tonsillar hypertrophy who had undergone a tonsillectomy. All procedures using human tonsil tissue or tissue-derived cells were conducted in accordance with guidelines approved by the Pusan National University Hospital Institutional Review Board. We utilized the cells that we verified the characteristics of T-MSCs by determining the proliferation, differentiation, and surface markers with Flow cytometry (FACS) as we previously reported [12].

### Purification and identification of T-MSCs-derived extracellular vesicles

T-MSCs were changed with the Exo-FBS™ Exosome-FBS (System Biosciences, CA, USA). Media supplement for an additional 48 h. After 48 hours, the culture medium of T-MSCs was obtained and centrifuged at 2,000 × g for 15 min to eliminate dead cells and cellular debris. The obtained medium underwent centrifugation at 10,000 × g for 60 min, and the supernatant was then filtrated with 0.22 μm filters to remove cellular debris and large particles further. Cell supernatants were collected and, after filtering by 0.22 μm filters, the supernatants were stored at −80°C and EV were isolated using an Exo Quick-TC kit (System Bioscience, CA, USA) according to the manufacturer’s instructions. Briefly, the cells were centrifuged at 3,000 g for 15 min to remove cells and cell debris. The supernatant was transferred to a sterile vessel, and an appropriate volume of Exo Quick-TC (1:5) was added. The samples were mixed before incubation overnight at 4°C for 24 hr. EV were isolated by centrifugation for 30 min at 1500 g. The common exosomal surface markers CD9, CD63, CD81 and HSP 70 were measured by western blotting with ExoAb Antibody Sampler Kit (BioCat, Heidelberg Germany).

### Intra/inter-assay variation of NanoSight measurements

The ultrastructure and size distribution of extracellular vesicles were analyzed by NanoSight NS500 instruments (Malvern Instruments, Amesbury, UK) respectively. The instruments were equipped with a 488 nm laser, a high sensitivity sCMOS camera and a syringe pump. There is no pre-treatment, we used to dilute in particle-free PBS (0.02 μm filtered) to obtain a concentration within the recommended measurement range 106 to 109 particles per ml. Experiment videos were analyzed using NTA 2.3 software (Malvern) after capture in script control mode (2 videos of 10 s per measurement). Samples were captured and analyzed by applying either identical or instrument settings [45].

### Dynamic light scattering of exosomes

The size of isolated extracellular vesicles was determined using dynamic light scattering using a Zetasizer Nano ZS DLS instrument (Malvern Instruments, UK) as previously described [46]. All experiments were performed in triplicate.

### Next generation sequencing library generation and microRNA sequencing

NGS libraries with isolated extracellular vesicles were generated with the TailorMix Micro RNA Sample Preparation version 2 protocol with isolated extracellular vesicles (SeqMatic LLC, Fremont, CA, USA). Briefly, 3′-adapter was ligated to the RNA sample and excess 3′-adapters were removed subsequently. 5′-adapter was then ligated to the 3′-adapter ligated samples, followed by first strand cDNA synthesis. cDNA library was amplified and barcoded via enrichment PCR. Final RNA library was size-selected on an 8% TBE polyacrylamide gel. Sequencing was performed on the Illumina NextSeq 500 platform at 1 × 75 bp single-end at SR50.

### LC-MS spectrometry proteomics

Half of the digested sample was analyzed by nano LC-MS/MS with a Waters NanoAcquity HPLC system interfaced to a ThermoFisher Q Exactive. Peptides were loaded on a trapping column and eluted over a 75 μm analytical column at 350 nL/min using a 2 hr reverse phase gradient; both columns were packed with Luna C18 resin (Phenomenex). The mass spectrometer was operated in data-dependent mode, with the Orbitrap operating at 70,000 FWHM and 17,500 FWHM for MS and MS/MS respectively. The fifteen most abundant ions were selected for MS/MS.

### Western blot

Western blotting was carried out as described previously. The protein concentration was determined using the BCA protein assay kit (Thermo Scientific Pierce). Total protein equivalents for each sample were separated by sodium dodecyl sulfate-polyacrylamide gel electrophoresis (SDS-PAGE) using 10% acrylamide gels as described by previously and transferred to PVDF membranes. The membrane was immediately placed into blocking buffer (1% non-fat milk) for 1 hr. The membrane was incubated with anti-AQP5 and α-amylase (Santa Cruz, 1:2000) at 4°C overnight and using anti-β-actin antibody (Santa Cruz, 1:2000) as an internal control. After three 10-min washes, the membranes were incubated with goat anti-rabbit IgG (1:10000 dilution) for 1 h at room temperature. Antibody labeling was detected using West-zol Plus and chemiluminescence Fluorchem™ SP (Alpha Innotech Corporation, San Leandro, CA, USA).

### Animal model establishment

Eighteen female Sprague–Dawley rats, aged nine weeks, were used in this study (Samtako, Osan, Korea). Each group was weight-matched at the beginning of the study. After a week for acclimatization, rats were randomly divided into three groups. Group I (*n* = 6, sham-operated rats, called HAM), group II (*n* = 6, ovariectomized surgery rats injected with saline on submandibular gland, called OVX), and group III (*n* = 6, ovariectomized surgery rats injected with extracellular vesicles on submandibular gland, called OVX+EXO). Rats were sacrificed 6 weeks after ovariectomy. For OVX surgery, the rats were anesthetized using isoflurane inhalation (3% dissolved in oxygen) and an incision made at the midline of the abdomen with the bilateral ovaries being revealed. In the OVX group, the ovaries were ligated and cut off bilaterally followed by the closure of the abdominal cavity. When ovariectomy was performed, extracellular vesicles extracted from 3 × 10^7^ cells diluted in saline were injected into the left submandibular gland by exposing the salivary glands immediately after ovariectomy surgery in rats.

### Plasma estradiol analysis

Concentrations of estradiol in serum were measured by rat-specific estradiol enzyme-linked immunosorbent (ELISA) assay plates coated with a biotin-conjugated binding protein kit purchased from Calbiotech (Spring Valley, CA, USA). A cardiac puncture was performed and the blood spun at 3000 rpm for 30 min. Plasma was separated from the blood collected during exsanguination, immediately frozen in liquid nitrogen, and then stored at −80°C.

### Staining and immunohistochemistry analysis

The submandibular gland was isolated from each rat and prepared for fixation overnight in 4% formalin and embedded with paraffin. Cross-sections were prepared for Hematoxylin and eosin, Masson’s trichrome staining and immunohistochemistry. For quantitative analyses of collagen I, TGF βI, AQP5 and α-amylase expression, de-paraffinized sections were incubated for 24 h at 4°C with the following primary antibodies: anti-collagen I, TGF βI and AQP5 (200 ug/mL) (Santa Cruz Biotechnology, Dallas, TA, USA), anti- α-amylase (1:400) (Abcam, Cambridge, UK). After the primary antibody was removed by rinsing, sections were incubated with goat-anti rabbit secondary antibodies (1:1000) (ENZO Biochem, NY, USA) for 1 h at room temperature) and following double-stained with DAB (3,3-diaminobenzidine). Incubation with phosphate-buffered saline supplemented with 1% bovine serum albumin instead of the primary antibody served as a negative control.

### Quantitative PCR

The submandibular gland RNA was extracted using the TRIzol system (Life Technologies, Rockville, MD, USA). A reverse transcription kit (Applied Biosystems, Foster City, CA, USA) was used to perform reverse transcription according to the manufacturer’s protocol. RNA concentration was measured with NanoDrop^®^ ND-1000 Spectrophotometers. The ratio of absorbance at 260 nm/280 nm is used to assess the purity of RNA. Quantitative PCR was performed according to the SYBR^®^ Green PCR protocol (Applied Biosystems, Foster city, CA, USA). Reaction conditions were: 10 min at 95°C (one cycle); 10 s at 95°C; and 30 s at 60°C (40 cycles). Gene-specific PCR products were continuously measured by an ABI PRISM 7900 HT Sequence Detection System (PE Applied Biosystems, Waltham, CT, USA). Primer sequences can be found in Table 1. Normalization consisted of using the differences between the cycle thresholds (delta CT) and the expression level for *GAPDH* to calculate the delta CT/target gene delta CT ratio.

**Table 1:** Primers.

**Gene**	**Direction**	**Sequence**
ERβ1	Forward	GCTTCGTGGAGCTCAGCCTG
	Reverse	AGGATCATGGCCTTGACACAGA
ERβ2	Forward	GAAGCTGAACCACCCAATGT
	Reverse	CAGTCCCACCATTAGCACCT
TNFα	Forward	GGTCAACCTGCCCAAGTACT
	Reverse	CTCCAAAGTAGACCTGCCCG
IFNγ	Forward	AGCCTAAGGAAGCGGAAAAG
	Reverse	GGCACACTCTCTACCCCAGA
IL-1β	Forward	CCCCACTTGAAGCAGATGACC
	Reverse	CCCTAAGTACTGGTAGTCCGC
IL-6	Forward	ATCTGCCCTTCAGGAACAGC
	Reverse	GAAGTAGGGAAGGCAGTGGC
Collagen I	Forward	CAGGATGCAGTCCCTGAAAT
	Reverse	GAGGTGGCCTAGGTGGTGTA
Collagen III	Forward	GGCCCTGTGTGTACTGGTCT
	Reverse	AGCATCAGAGGGAGTGAGGA
TGFβI	Forward	GACGTTCGCCATAACCAAGT
	Reverse	CTGCAGGTTCTCAATGCAAA
TGFβII	Forward	CCAATCACGCAATAGTTCTGG
	Reverse	CGCTGTATCGTATGGCGAT
AQP3	Forward	AATTGTCTGGAGCCCACTTG
	Reverse	CAGCTTGATCCAGGGCTCTC
AQP5	Forward	CATGAACCCAGCCCGATCTT
	Reverse	AGAAGACCCAGTGAGAGGGG
GAPDH	Forward	ATCAAGAAGGTGGTGAAGCA
	Reverse	AAGGTGGAAGAATGGGAGTTG

### Statistical analysis

Unless otherwise noted, all quantitative data were reported as the mean standard error of the mean from at least three parallel repeats. Two-way analysis of variance (ANOVA) was used to determine significant differences between groups in which *P* < 0.05 was considered statistically significant.

### Data availability

All the data are available in the main text and supplemental materials.

## Supplementary Materials

Supplementary Video 1

Supplementary Video 2

## References

[1] Schneider B, van Trotsenburg M, Hanke G, Bigenzahn W, Huber J. Voice impairment and menopause. Menopause. 2004; 11:151–8. 10.1097/01.gme.0000094192.24934.46 15021444

[2] Suri V, Suri V. Menopause and oral health. J Midlife Health. 2014; 5:115–20. 10.4103/0976-7800.141187 25316996 PMC4195183

[3] Chi AC, Neville BW, Krayer JW, Gonsalves WC. Oral manifestations of systemic disease. Am Fam Physician. 2010; 82:1381–8. 21121523

[4] Meurman JH, Tarkkila L, Tiitinen A. The menopause and oral health. Maturitas. 2009; 63:56–62. 10.1016/j.maturitas.2009.02.009 19324502

[5] Caplan AI. Adult mesenchymal stem cells for tissue engineering versus regenerative medicine. J Cell Physiol. 2007; 213:341–7. 10.1002/jcp.21200 17620285

[6] Kolf CM, Cho E, Tuan RS. Mesenchymal stromal cells. Biology of adult mesenchymal stem cells: regulation of niche, self-renewal and differentiation. Arthritis Res Ther. 2007; 9:204. 10.1186/ar2116 17316462 PMC1860068

[7] An HY, Shin HS, Choi JS, Kim HJ, Lim JY, Kim YM. Adipose Mesenchymal Stem Cell Secretome Modulated in Hypoxia for Remodeling of Radiation-Induced Salivary Gland Damage. PLoS One. 2015; 10:e0141862. 10.1371/journal.pone.0141862 26529411 PMC4631328

[8] Ryu KH, Cho KA, Park HS, Kim JY, Woo SY, Jo I, Choi YH, Park YM, Jung SC, Chung SM, Choi BO, Kim HS. Tonsil-derived mesenchymal stromal cells: evaluation of biologic, immunologic and genetic factors for successful banking. Cytotherapy. 2012; 14:1193–202. 10.3109/14653249.2012.706708 22900958

[9] Seo Y, Shin TH, Ahn JS, Oh SJ, Shin YY, Yang JW, Park HY, Shin SC, Kwon HK, Kim JM, Sung ES, Park GC, Lee BJ, Kim HS. Human Tonsil-Derived Mesenchymal Stromal Cells Maintain Proliferating and ROS-Regulatory Properties via Stanniocalcin-1. Cells. 2020; 9:636. 10.3390/cells9030636 32155780 PMC7140534

[10] Lim YS, Lee JC, Lee YS, Lee BJ, Wang SG. Growth Inhibitory Effect of Palatine Tonsil-derived Mesenchymal Stem Cells on Head and Neck Squamous Cell Carcinoma Cells. Clin Exp Otorhinolaryngol. 2012; 5:86–93. 10.3342/ceo.2012.5.2.86 22737289 PMC3380118

[11] Shin SC, Seo Y, Park HY, Jung DW, Shin TH, Son H, Kim YK, Lee JC, Sung ES, Jang JY, Kim HS, Lee BJ. Regenerative potential of tonsil mesenchymal stem cells on surgical cutaneous defect. Cell Death Dis. 2018; 9:183. 10.1038/s41419-017-0248-4 29416004 PMC5833728

[12] Park GC, Kim HS, Park HY, Seo Y, Kim JM, Shin SC, Kwon HK, Sung ES, Lee JC, Lee BJ. Tensin-3 Regulates Integrin-Mediated Proliferation and Differentiation of Tonsil-Derived Mesenchymal Stem Cells. Cells. 2019; 9:89. 10.3390/cells9010089 31905841 PMC7017379

[13] Akyurekli C, Le Y, Richardson RB, Fergusson D, Tay J, Allan DS. A systematic review of preclinical studies on the therapeutic potential of mesenchymal stromal cell-derived microvesicles. Stem Cell Rev Rep. 2015; 11:150–60. 10.1007/s12015-014-9545-9 25091427

[14] EL Andaloussi S, Mäger I, Breakefield XO, Wood MJ. Extracellular vesicles: biology and emerging therapeutic opportunities. Nat Rev Drug Discov. 2013; 12:347–57. 10.1038/nrd3978 23584393

[15] Breitbach M, Bostani T, Roell W, Xia Y, Dewald O, Nygren JM, Fries JW, Tiemann K, Bohlen H, Hescheler J, Welz A, Bloch W, Jacobsen SE, Fleischmann BK. Potential risks of bone marrow cell transplantation into infarcted hearts. Blood. 2007; 110:1362–9. 10.1182/blood-2006-12-063412 17483296

[16] Yoon YS, Park JS, Tkebuchava T, Luedeman C, Losordo DW. Unexpected severe calcification after transplantation of bone marrow cells in acute myocardial infarction. Circulation. 2004; 109:3154–7. 10.1161/01.CIR.0000134696.08436.65 15197139

[17] Berglund AK, Fortier LA, Antczak DF, Schnabel LV. Immunoprivileged no more: measuring the immunogenicity of allogeneic adult mesenchymal stem cells. Stem Cell Res Ther. 2017; 8:288. 10.1186/s13287-017-0742-8 29273086 PMC5741939

[18] Du YM, Zhuansun YX, Chen R, Lin L, Lin Y, Li JG. Mesenchymal stem cell exosomes promote immunosuppression of regulatory T cells in asthma. Exp Cell Res. 2018; 363:114–20. 10.1016/j.yexcr.2017.12.021 29277503

[19] Cho KA, Park M, Kim YH, Ryu KH, Woo SY. Poly I:C primes the suppressive function of human palatine tonsil-derived MSCs against Th17 differentiation by increasing PD-L1 expression. Immunobiology. 2017; 222:394–8. 10.1016/j.imbio.2016.08.012 27594385

[20] Lou G, Chen Z, Zheng M, Liu Y. Mesenchymal stem cell-derived exosomes as a new therapeutic strategy for liver diseases. Exp Mol Med. 2017; 49:e346. 10.1038/emm.2017.63 28620221 PMC5519012

[21] Volarevic V, Markovic BS, Gazdic M, Volarevic A, Jovicic N, Arsenijevic N, Armstrong L, Djonov V, Lako M, Stojkovic M. Ethical and Safety Issues of Stem Cell-Based Therapy. Int J Med Sci. 2018; 15:36–45. 10.7150/ijms.21666 29333086 PMC5765738

[22] Eirin A, Zhu XY, Puranik AS, Tang H, McGurren KA, van Wijnen AJ, Lerman A, Lerman LO. Mesenchymal stem cell-derived extracellular vesicles attenuate kidney inflammation. Kidney Int. 2017; 92:114–24. 10.1016/j.kint.2016.12.023 28242034 PMC5483390

[23] Suzuki E, Fujita D, Takahashi M, Oba S, Nishimatsu H. Stem cell-derived exosomes as a therapeutic tool for cardiovascular disease. World J Stem Cells. 2016; 8:297–305. 10.4252/wjsc.v8.i9.297 27679686 PMC5031891

[24] Kim JM, Shin SC, Park GC, Lee JC, Jeon YK, Ahn SJ, Thibeault S, Lee BJ. Effect of sex hormones on extracellular matrix of lamina propria in rat vocal fold. Laryngoscope. 2020; 130:732–40. 10.1002/lary.28086 31180590

[25] Abd El-Haleem MR, Selim AO, Attia GM. Bone marrow-derived mesenchymal stem cells ameliorate parotid injury in ovariectomized rats. Cytotherapy. 2018; 20:204–17. 10.1016/j.jcyt.2017.10.003 29254763

[26] El-Naseery NI, Elewa YHA, Ichii O, Kon Y. An experimental study of menopause induced by bilateral ovariectomy and mechanistic effects of mesenchymal stromal cell therapy on the parotid gland of a rat model. Ann Anat. 2018; 220:9–20. 10.1016/j.aanat.2018.06.006 30040990

[27] Zhang X, Wu S, Naccarato T, Prakash-Damani M, Chou Y, Chu CQ, Zhu Y. Regeneration of hyaline-like cartilage in situ with SOX9 stimulation of bone marrow-derived mesenchymal stem cells. PLoS One. 2017; 12:e0180138. 10.1371/journal.pone.0180138 28666028 PMC5493350

[28] Zhu B, Liu W, Zhang H, Zhao X, Duan Y, Li D, Jin Y. Tissue-specific composite cell aggregates drive periodontium tissue regeneration by reconstructing a regenerative microenvironment. J Tissue Eng Regen Med. 2017; 11:1792–805. 10.1002/term.2077 26455905

[29] Kelm JM, Breitbach M, Fischer G, Odermatt B, Agarkova I, Fleischmann BK, Hoerstrup SP. 3D microtissue formation of undifferentiated bone marrow mesenchymal stem cells leads to elevated apoptosis. Tissue Eng Part A. 2012; 18:692–702. 10.1089/ten.TEA.2011.0281 21988679

[30] Jung JW, Kwon M, Choi JC, Shin JW, Park IW, Choi BW, Kim JY. Familial occurrence of pulmonary embolism after intravenous, adipose tissue-derived stem cell therapy. Yonsei Med J. 2013; 54:1293–6. 10.3349/ymj.2013.54.5.1293 23918585 PMC3743204

[31] Keerthikumar S, Chisanga D, Ariyaratne D, Al Saffar H, Anand S, Zhao K, Samuel M, Pathan M, Jois M, Chilamkurti N, Gangoda L, Mathivanan S. ExoCarta: A Web-Based Compendium of Exosomal Cargo. J Mol Biol. 2016; 428:688–92. 10.1016/j.jmb.2015.09.019 26434508 PMC4783248

[32] Yeo RW, Lai RC, Zhang B, Tan SS, Yin Y, Teh BJ, Lim SK. Mesenchymal stem cell: an efficient mass producer of exosomes for drug delivery. Adv Drug Deliv Rev. 2013; 65:336–41. 10.1016/j.addr.2012.07.001 22780955

[33] Hu GW, Li Q, Niu X, Hu B, Liu J, Zhou SM, Guo SC, Lang HL, Zhang CQ, Wang Y, Deng ZF. Exosomes secreted by human-induced pluripotent stem cell-derived mesenchymal stem cells attenuate limb ischemia by promoting angiogenesis in mice. Stem Cell Res Ther. 2015; 6:10. 10.1186/scrt546 26268554 PMC4533800

[34] Wang R, Xu B. TGF-β1-modified MSC-derived exosomal miR-135b attenuates cartilage injury via promoting M2 synovial macrophage polarization by targeting MAPK6. Cell Tissue Res. 2021; 384:113–27. 10.1007/s00441-020-03319-1 33404840

[35] Xia C, Zeng Z, Fang B, Tao M, Gu C, Zheng L, Wang Y, Shi Y, Fang C, Mei S, Chen Q, Zhao J, Lin X, et al. Mesenchymal stem cell-derived exosomes ameliorate intervertebral disc degeneration via anti-oxidant and anti-inflammatory effects. Free Radic Biol Med. 2019; 143:1–15. 10.1016/j.freeradbiomed.2019.07.026 31351174

[36] Zhao X, Zhao Y, Sun X, Xing Y, Wang X, Yang Q. Immunomodulation of MSCs and MSC-Derived Extracellular Vesicles in Osteoarthritis. Front Bioeng Biotechnol. 2020; 8:575057. 10.3389/fbioe.2020.575057 33251195 PMC7673418

[37] Kwon HK, Kim JM, Shin SC, Sung ES, Kim HS, Park GC, Cheon YI, Lee JC, Lee BJ. The mechanism of submandibular gland dysfunction after menopause may be associated with the ferroptosis. Aging (Albany NY). 2020; 12:21376–90. 10.18632/aging.103882 33159020 PMC7695378

[38] Kim J, Lee C, Shin Y, Wang S, Han J, Kim M, Kim JM, Shin SC, Lee BJ, Kim TJ, Jung Y. sEVs from tonsil-derived mesenchymal stromal cells alleviate activation of hepatic stellate cells and liver fibrosis through miR-486-5p. Mol Ther. 2021; 29:1471–86. 10.1016/j.ymthe.2020.12.025 33348053 PMC8058446

[39] Luo F, Sun Z, Han Q, Xue C, Bai C. Effect of Human Hepatocellular Carcinoma HepG2 Cell-derived Exosome on the Differentiation of Mesenchymal Stem Cells and Their Interaction. Zhongguo Yi Xue Ke Xue Yuan Xue Bao. 2017; 39:312–7. 28695799 10.3881/j.issn.1000-503X.2017.03.003

[40] Lou G, Chen L, Xia C, Wang W, Qi J, Li A, Zhao L, Chen Z, Zheng M, Liu Y. MiR-199a-modified exosomes from adipose tissue-derived mesenchymal stem cells improve hepatocellular carcinoma chemosensitivity through mTOR pathway. J Exp Clin Cancer Res. 2020; 39:4. 10.1186/s13046-019-1512-5 31898515 PMC6941283

[41] Zou X, Gu D, Xing X, Cheng Z, Gong D, Zhang G, Zhu Y. Human mesenchymal stromal cell-derived extracellular vesicles alleviate renal ischemic reperfusion injury and enhance angiogenesis in rats. Am J Transl Res. 2016; 8:4289–99. 27830012 PMC5095321

[42] Li T, Yan Y, Wang B, Qian H, Zhang X, Shen L, Wang M, Zhou Y, Zhu W, Li W, Xu W. Exosomes derived from human umbilical cord mesenchymal stem cells alleviate liver fibrosis. Stem Cells Dev. 2013; 22:845–54. 10.1089/scd.2012.0395 23002959 PMC3585469

[43] Tian MJ, Tu ZH, Hu R, Zhu XX. [Intervention of Yangfei Ziyin Decoction on Sjogren's Syndrome Model Mice]. Zhongguo Zhong Xi Yi Jie He Za Zhi. 2016; 36:63–8. 26955680

[44] Yamamura Y, Motegi K, Kani K, Takano H, Momota Y, Aota K, Yamanoi T, Azuma M. TNF-α inhibits aquaporin 5 expression in human salivary gland acinar cells via suppression of histone H4 acetylation. J Cell Mol Med. 2012; 16:1766–75. 10.1111/j.1582-4934.2011.01456.x 21973049 PMC3822690

[45] Kestens V, Bozatzidis V, De Temmerman PJ, Ramaye Y, Roebben G. Validation of a particle tracking analysis method for the size determination of nano- and microparticles. J Nanopart Res. 2017; 19:271. 10.1007/s11051-017-3966-8 28824287 PMC5543194

[46] Lyu TS, Ahn Y, Im YJ, Kim SS, Lee KH, Kim J, Choi Y, Lee D, Kang E, Jin G, Hwang J, Lee SI, Cho JA. The characterization of exosomes from fibrosarcoma cell and the useful usage of Dynamic Light Scattering (DLS) for their evaluation. PLoS One. 2021; 16:e0231994. 10.1371/journal.pone.0231994 33497388 PMC7837462

